# Influence of Hospitalization upon Diagnosis on the Risk of Tuberculosis Clustering

**DOI:** 10.4084/MJHID.2013.071

**Published:** 2013-11-20

**Authors:** Giuseppe Lapadula, Fabio Zanini, Luigi Codecasa, Fabio Franzetti, Maurizio Ferrarese, Manuela Carugati, Ester Mazzola, Consuelo Schiroli, Davide Motta, Diego Iemmi, Andrea Gori

**Affiliations:** 1 Clinic of Infectious Diseases, AO San Gerardo, Monza (Italy); 2 Luigi Sacco Hospital, University of Milan (Italy); 3 Villa Marelli, AO Cà Granda Niguarda, Milan (Italy); 4 Laboratory of Microbiology, AO Cà Granda Niguarda, Milan (Italy); 5 Clinic for Infectious and Tropical Diseases, Spedali Civili, University of Brescia, Brescia (Italy)

## Abstract

**Setting:**

Culture-positive tuberculosis (TB) diagnosed in the metropolitan area of Milan (Italy) over a 5-year period (1995–1999).

**Objective:**

To assess the impact of short-course hospitalization upon diagnosis on the overall risk of TB clustering.

**Design:**

Restriction fragment length polymorphism profiles with a similarity of 100% defined a cluster. Uni- and multivariable logistic regression models were performed to assess factors associated with clustering.

**Results:**

Among 1139 patients, 392 (34.4%) were hospitalized before or soon after diagnosis, 405 (35.6%) received domiciliary treatment since the diagnosis and 392 (30%) had no information about initial clinical management. One hundred fifteen molecular clusters involving 363 patients were identified. Using multivariable analysis, hospitalization was not significantly associated with clustering (OR 1.06, 95%CI 0.75–1.50, p=0.575). Subjects aged >65 years old (OR 0.60; 95CI%:0.37–0.95; p=0.016) and non-Italian born patients (OR 0.56; 95%CI:0.41–0.76; p<0.001) were running a lower risk of clustering. Conversely, HIV co-infected patients (OR 1.88, 95%CI:1.20–2.95, p=0.006) and those with MDR TB (OR 2.50, 95%CI:1.46–4.25, p=0.001) were significantly more likely to be involved in clusters.

**Conclusion:**

In our cohort, domiciliary treatment was not associated with TB clustering. Expanding domiciliary treatment upon diagnosis appears as an advisable measure to reduce unnecessary costs for the health care system.

## Introduction

Tuberculosis (TB) is still a leading cause of illness and death, not only in developing country but also in industrialized settings. The 4-drugs standard regimen is pivotal in TB treatment and is associated with a rapid reduction in infectivity. During the ‘60s, it was first demonstrated that segregation in sanatoria did not reduce TB transmission among family close contacts of patients. Actually, the incidence of active tuberculosis and tuberculosis infection was shown to be similar among contacts of patients treated at home or treated in sanatorium.^1^,^2^ On the other hand, hospitalization of TB patients has led, in past years, to nosocomial epidemics (often due to multidrug resistant strains) involving healthcare workers and HIV-infected subjects.^3^–^7^ More recently, ongoing multi-drug resistant TB transmission within Latvian hospitals was demonstrated combining molecular and epidemiological methods (whilst no epidemiological link was found among clustered drug-susceptible TB cases).^8^ Basing on these evidences, domiciliary treatment is considered safe and appropriate in the vast majority of cases. Nonetheless, the infectivity is unlikely to disappear immediately after therapy is started and hospitalization with strict isolation measures during the early phase of treatment remains a common practice in some high-income settings, where isolation areas and controlled ventilation systems are available. Whether this approach can further reduce the risk of disease dissemination in countries with low TB prevalence merits to be investigated further. The use of molecular epidemiology can be extremely useful in this respect, given its ability to identify unapparent pathway of transmission and to define clusters of patients epidemiologically linked.^9^

## Patients and Methods

### Study population

All culture-confirmed cases of tuberculosis observed between 1995 and 1999 in the metropolitan area of Milan with an available Restriction Fragment Length Polymorphism (RFLP) IS6110 analysis were considered in the study. The study was conducted in the urban and suburban area of Milan, comprising an area of more than 5,000,000 inhabitants.

Demographics, clinical characteristics and epidemiological data were obtained from “Istituto Villa Marelli”, Milan (the regional reference centre for mycobacterial infection, collecting all strains isolated in the metropolitan area of Milan). Clinical data were integrated with those of patients hospitalized with a diagnosis of tuberculosis at “L.Sacco Hospital” (reference hospital for infectious diseases in Milan). Data were then cross-checked and further integrated with the electronic regional register for infectious diseases notification of Lombardia.

Patients were categorized according to the clinical management during the early phase of treatment, distinguishing two groups: those who were hospitalized before or soon after the diagnosis and those who received domiciliary treatment since the diagnosis.

### Molecular epidemiology methods

RFLP genotyping was performed in the mycobacterial laboratory of the Infectious Diseases Clinic of Sacco Hospital, Milan. Extraction of DNA from mycobacterial strains and DNA fingerprinting with IS6110 as a probe were performed as follows: after harvesting and killing of mycobacteria previously cultured on a Lowenstein-Jensen medium, 5 μg of genomic DNA was digested with PvuII. DNA fragments were separated by electrophoresis on agarose gels, denatured, and blotted onto nylon membrane by Southern Blotting. Hybridization was performed overnight at 42°C on PvuII-restricted genomic DNA with a chemiluminescence-labeled 521-bp IS6110 fragment and pgrs on IndiIII-restricted genomic DNA. Presence of DNA probe was assessed with ECL detection system (Amersham), and dendrogram of similarity were obtained comparing RFLP fingerprint with Bionumerics software (Applied Maths, Sint-Martens-Latem, Belgium).

A group of two or more *M. tuberculosis* strains, isolated from different patients, with a RFLP pattern similarity of 100% was considered as a cluster, thus epidemiologically meaning a recent transmission (not necessarily direct). Other RFLP fingerprints were considered ungrouped and classified as sporadic cases. Cluster composed by low-copy-number strains, i.e. strains with a number of IS6110 copies ≤5, were further studied using 12-loci mycobacterial interspersed repetitive units (MIRU) analysis, as described elsewhere,^10^,^11^ in order to confirm or exclude the clustering. Clusters involving more than 5 patients were defined as “macroclusters”.

### Statistical analysis

Uni- and multivariable logistic regression analyses were performed to assess factors associated with clustering. The following factors were tested: age, gender, country of birth, hospitalization at diagnosis, site of infection (pulmonary *versus* extrapulmonary or disseminated), sputum smear examination, case definition (new *versus* relapse/failure), year of diagnosis, drug-resistance, HIV co-infection. A separate category was created for any missing data. All variables associated with the outcome with a P <0.20 at univariate analysis and hospitalization at diagnosis were included in the multivariable analysis.

Sensitivity analyses were conducted excluding three large clusters, related to nosocomial epidemics among HIV-co infected subjects, and excluding MDR-TB, to ensure that these patient populations were not overtly influencing the results.

The analysis was carried out using SAS/STAT statistic software v8.2 (SAS Institute Inc., Cary, USA) and all P-values presented are two-sided. A P-value <0.05 indicated conventional statistical significance.

## Results

### Patients characteristics and clustering

Between January 1^st^ 1995 and December 31^st^ 1999, 1139 cases of culture-confirmed TB were reported in the metropolitan area of Milan and had an available clinical isolate for genotypic RFLP analysis. Their characteristics are shown in Table 1. Among enrolled patients, 392 (34.4%) had been hospitalized before or soon after diagnosis and 405 (35.6%) had received domiciliary treatment since the diagnosis. In 392 (30%) cases, the information about initial clinical management could not be retrieved.

At RFLP analysis, 363 (31.9%) isolates were involved in 115 clusters, whereas 776 (68.1%) isolates had a sporadic pattern. The rate of genetic diversity was therefore 0.78 (891 different patterns among 1139 patients). The dimension of the clusters ranged from 2 to 13 subjects and 104 patients (9.1%) belonged to 13 macroclusters.

### Impact of hospitalization

The majority of clusters (76/115 = 66.1%) involved at least one hospitalized patient and 26/115 (22.6%) were composed only by hospitalized patients. The proportion of patients hospitalized at diagnosis remained stable over time, ranging from 35.9% in 1995 to 36.4% in 1999 (Chi-square for trend P=0.744), whilst the proportion of patients involved in clusters decreased from 46.4% in 1995 to 26.4% in 1999 (Chi square for trend P<0.001).

Using univariate logistic regression analysis, hospitalization was not significantly associated with clustering (OR 1.23, 95%CI 0.91–1.67, p=0.368). Conversely, Italian patients, HIV co-infected subjects and those diagnosed with TB in earlier years were running a significantly higher risk of clustering (see Table 2).

Using multivariable analysis, hospitalized patients were confirmed not to run a higher risk of clustering than patients who received domiciliary treatment since the diagnosis (OR 1.06, 95%CI 0.75–1.50, p=0.575), independently of other possible confounders. Conversely, HIV co-infection (OR 1.88, 95%CI 1.20–2.95, p=0.006) and MDR TB (OR 2.50, 95%CI 1.46–4.25, p=0.001) were significantly associated with risk of clustering. Moreover, subjects aged >65 years old (OR 0.60; 95CI% 0.37–0.95; p=0.016) and non-Italian born patients (OR 0.56; 95%CI 0.41–0.76; p<0.001) were less likely to be involved in clusters.

### Sensitivity analysis

A sensitivity analysis was carried out excluding three large clusters (25 patients), which were related to nosocomial epidemics of multi-drug resistant TB occurred in the hospitals of Milan city area between 1993 and 1997 and mainly involving Italian HIV-co infected subjects.

After the exclusion of these patients, hospitalization was not significantly associated with clustering (unadjusted OR 1.14, 95%CI 0.84–1.55, p=0.887). Using multivariable analysis, hospitalization was confirmed not to be significantly associated with clustering (OR 1.07, 95%CI 0.76–1.52, p=0.772), independently of age, nationality, year of diagnosis, TB drug susceptibility and HIV-co infection.

Similar results were obtained in a sensitivity analysis performed only on 1059 patients with drug-susceptible TB (data not shown).

## Discussion

Since the introduction of effective chemotherapy, domiciliary treatment was demonstrated to be as effective as hospital treatment. A large randomized controlled trial conducted in Madras (presently known as Chennai, India) demonstrated that domiciliary treatment was not associated with an increased risk of transmission to the household contacts of patients suffering from TB, as compared with a 1-year segregation in sanatorium.^1^,^2^ The study, however, was conducted in a setting of high prevalence of TB, as suggested by the very low proportion (30%) of household contacts with negative tuberculin skin testing at baseline and by the high rate (>20%) of skin test conversion before discharge from sanatorium of the index case.^1^ Moreover, the treatment used in the study was far from being as effective as today, given the high proportion of patients with positive sputum cultures after 1 year of treatment and the high rate of relapse in 5 years.^2^ Therefore, the findings of this study are not immediately generalizable to the present situation of many high-income countries. Subsequent studies conducted in high-income settings during the 70’s demonstrated that a policy of early hospital discharge was not associated with TB transmission, thus suggesting that patients can safely be discharged after 2 weeks of treatment, even if sputum smear examination is still positive.^12^–^14^ However, the comparison of the classic Madras study between institutional and domiciliary treatment since the diagnosis has never been repeated in countries with low TB prevalence, where strict respiratory isolation of patients (single rooms, controlled ventilation system with negative pressure) during the first days of therapy is possible.

In our cohort, all the analyses consistently demonstrated that domiciliary treatment was not associated with TB clustering. Short-course hospitalization was not associated with any advantage in terms of reduced TB clustering. This finding was confirmed even when factors known to influence TB transmission, such as HIV co-infection 15,16 and being local-born 15,17–20, were taken into account. Moreover, a substantial number of the identified clusters of transmission were mainly or completely composed by hospitalized patients, thus suggesting that nosocomial TB transmission could be an issue. Taken all together, our findings support the indication to limit hospitalization to patient for whom there is a clinical indication to hospital treatment, such as patient conditions, the risk of low adherence, the likelihood of infection with multidrug-resistant organism or cohabitation with subjects with high-risk of TB disease (i.e., children <4 years old).

A high rate of clustering was, however, demonstrated and about one third of patients were shown to be involved in clusters. This finding is consistent with the hypothesis that active transmission is a common phenomenon also in countries with low TB prevalence, as previously reported.^21^,^22^ Much of the transmission is likely to have occurred in the period preceding the diagnosis, when the patients were unaware of any infectiousness, or to have been caused by patients with low adherence to prescribed treatment. Prevention of TB transmission should be therefore focused on these two issues. Human and economical resources should be devoted to early diagnosis and prompt treatment of new TB cases and to active tracing of patients missing scheduled visits.

Despite current national and international guidelines support out-patient management, a significative proportion of the patients in our cohort was admitted to hospital. This finding confirms the results from a previous national survey, demonstrating that the majority of TB patients in Italy are hospitalized.^23^ An excess in hospitalization is likely to lead to unnecessary costs for the Health National System and to a possible higher risk of nosocomial transmission. Therefore, a change in this policy is advisable, because routine hospitalization of all patients with pulmonary TB is still practiced in many points of care. Updates of hospital guidelines and educational programmes addressed to health staff dealing with TB are constantly needed.

The present study has some limitations that should be acknowledged. First, no information was collected on standard epidemiological surveillance (namely, on the rate of tuberculin skin test conversion among contacts). This may have led to underestimate the risk of TB transmission, because most infections are followed by a variable latency period. However, molecular clustering can reasonably approximate epidemiologic clustering and recent transmission in low-incidence populations.^9^ Moreover, since most immunocompetent individuals infected with *M. tuberculosis* remain disease-free during the course of their lives, focusing the study only on active cases of TB can be more informative about the effect of public health policy on the TB epidemic. Second, given the non-randomized, observational nature of the study, we cannot exclude that patients receiving domiciliary treatment were those judged to be less infectious. Nonetheless, the results of our study were confirmed even adjusting for the measurable markers of infectiousness (such as sputum smear positivity).

In any case, a strength of the present study is the large number of the patient involved, which allowed to detect clusters that, in a narrower study, could have resulted to be smaller or even unnoticed. In addition, to our knowledge this is the first time that molecular epidemiology is used to evaluate the impact of the isolation policy on the risk of TB transmission. Such a novel approach could be applied in other settings, in order to evaluate the effect of changes in health care policy on *M. tuberculosis* circulation.

## Conclusion

Our results suggest that hospitalization of TB patients is not associated with a reduction in clustering risk. Expanding domiciliary treatment upon diagnosis is an advisable measure to reduce unnecessary costs for the health care system.

## Figures and Tables

**Table 1:** Patients’ characteristics (study population *versus* general population).

Characteristics	Study population N=1139
Age [ Mean (SD) ]*	45 (19.9)

Female gender [ N (%) ]	425 (37.4)

Country of Birth	
Italy	773 (67.9)
Other	366 (32.1)
North Africa	39 (3.4)
Sub-Saharian Africa	55 (4.8)
South America	108 (9.5)
Asia	50 (4.4)
Eastern Europe	17 (1.5)
Other	3 (0.3)
Unknown not-Italian	94 (8.2)

Year of diagnosis	
1995	153 (13.4)
1996	258 (22.6)
1997	160 (14)
1998	307 (26.9)
1999	261 (22.9)

Clinical management [ N (%) ]	
Hospitalization	392 (34.4)
Domiciliary treatment	405 (35.6)
Unknown	342 (30)

Case definition [ N (%) ]	
New	969 (85.1)
Failure/relapse	26 (2.3)
Unknown	144 (12.6)

Site of infection [ N (%) ]	
Pulmonary	695 (61)
Extrapulmonary	138 (12.1)
Pulmonary & extrapulmonary	32 (2.8)
Unknown	274 (24.1)

AFB smear** [ N (%) ]	
Positive	332 (45.7)
Negative	147 (20.2)
Unknown	248 (34.1)

Multi drug resistance [ N (%) ]	73 (6.4)

HIV-Ab positivity [ N (%) ]	119 (10.4)

AFB, acid-fast bacilli; HIV-Ab, Human Immunodeficiency Virus Antibody; SD, standard deviation; N, number. Age at diagnosis available for 670 patients among the study population and for 768 patients among the general population. Sputum smear microscopic examination for acid-fast bacilli available for 727 patients with pulmonary involvement among the study population and for 821 patients with pulmonary involvment among the general population.

**Table 2:** Risk of clustering. Uni- and multivariable logistic regression analyses

	Univariable			Multivariable		
Variables	OR	95%CI	P	OR	95%CI	P
Age						
< 65 years old	1	-		1	-	
>65 years old	0.62	0.40–0.94	0.018	0.60	0.37–0.95	0.016
Unknown	1.01	0.78–1.32	0.083	1.08	0.69–1.67	0.133

Female gender	0.80	0.62–1.04	0.102		Dropped	

Non-Italian born	0.62	0.47–0.82	0.001	0.56	0.41–0.76	<0.001

Pulmonary involvement				Not entered		
No	1	-				
Yes	1.22	0.81–1.83	0.830			
Unknown	1.40	0.89–2.19	0.150			

Case classification				Not entered		
New Case	1	-				
Relapse or Failure	1.59	0.72–3.50	0.265			
Unknown	1.02	0.70–1.48	0.425			

AFB smear				Not entered		
Negative	1	-				
Positive	1.08	0.71–1.64	0.733			
Unknown	1.04	0.67–1.62	0.992			

Hospitalization						
No	1	-		1		
Yes	1.23	0.91–1.67	0.368	1.06	0.75–1.50	0.575
Unknown	1.20	0.88–1.64	0.577	0.91	0.59–1.41	0.583

Multi drug resistance	3.55	2.18–5.78	<0.001	2.50	1.46–4.25	0.001

HIV-Ab positivity	2.50	1.70–3.67	<0.001	1.88	1.20–2.95	0.006

Year of diagnosis						
1999	1	-		1	-	
1995	2.41	1.58–3.67	<0.001	1.75	1.12–2.73	0.034
1996	1.25	0.85–1.83	0.365	1.13	0.76–1.67	0.370
1997	1.81	1.18–2.75	0.069	1.76	1.15–2.71	0.022
1998	0.98	0.67–1.43	0.003	0.93	0.63–1.36	0.012

AFB, acid-fast bacilli; CI, confidence interval; HIV-Ab, Human Immunodeficiency Virus Antibody; OR, odds ratio. Odds ratios in multivariable model are adjusted for the other variable in the model. Association between clustering and sputum smear microscopic examination for acid-fast bacilli assessed only for patients with pulmonary involvement (N=727)
